# Assessing Cognitive-Motor Interference in Military Contexts: Validity and Reliability of Two Dual-tasking Tests

**DOI:** 10.1093/milmed/usad048

**Published:** 2023-04-18

**Authors:** Chiara Gattoni, Borja Martinez-Gonzalez, Caroline Li, Samuele Maria Marcora

**Affiliations:** School of Sport and Exercise Sciences, University of Kent, Canterbury, Kent CT2 7NH, UK; School of Sport and Exercise Sciences, University of Kent, Canterbury, Kent CT2 7NH, UK; Department of Biomedical and Neuromotor Sciences, University of Bologna, Centro Sportivo Universitario Record, Bologna 40127, Italy; School of Computing, University of Kent, Chatham Maritime, Kent ME4 4AG, UK; School of Sport and Exercise Sciences, University of Kent, Canterbury, Kent CT2 7NH, UK; Department of Biomedical and Neuromotor Sciences, University of Bologna, Centro Sportivo Universitario Record, Bologna 40127, Italy

## Abstract

**Introduction:**

Cognitive-motor interference is the decrease in cognitive performance and/or physical performance occurring when a cognitive task and a physical task are performed concurrently (dual task) compared to when they are performed in isolation (single task). The aim of this study was to investigate the construct validity and test–retest reliability of two cognitive-motor interference tests in military contexts.

**Materials and Methods:**

Twenty-two soldiers, officers, and cadets performed a 10-min loaded marching, a 10-min Psychomotor Vigilance Task, and the two tasks combined (visit 1). During visit 2, a 5-min running time trial, a 5-min Word Recall Task, and the two tasks combined. These tests were repeated by 20 participants after 2 weeks (visits 3 and 4).

**Results:**

Significant impairments were shown on both running distance (*P* < .001) and number of words recalled (*P* = .004) in the dual-task condition compared to the single-task condition. Significantly shorter step length (*P* < .001) and higher step frequency (*P* < .001) were found during the loaded marching in the dual-task condition compared to the single-task condition. No significant differences were observed in mean reaction time (*P* = .402) and number of lapses (*P* = .479) during the Psychomotor Vigilance Task. Good-to-excellent reliability was found for all the cognitive and physical variables in both single- and dual-task conditions, except for the number of lapses.

**Conclusion:**

These findings suggest that the Running + Word Recall Task test is a valid and reliable dual-tasking test that could be used to assess cognitive-motor interference in military contexts.

## INTRODUCTION

In military contexts, the ability to perform more than one task simultaneously (i.e., multitasking performance) is essential.^1^ However, errors and other performance impairments are often inevitable because of the higher workload that multitasks induce compared to single tasks, even in the most expert individuals.^2^ Different strategies have been proposed to improve the multitasking performance of military personnel, such as decreasing task overload, enhancing multitasking skills through specific occupational training, and employing individuals with superior multitasking abilities.^1^ The evaluation of these strategies requires valid and reliable measures. The best-known measure of multitasking performance in military contexts is the Multi-Attribute Task Battery-II developed by the National Aeronautics and Space Administration.^3^ However, the Multi-Attribute Task Battery-II does not include a physical task and does not induce significant physiological stress. The inclusion of a physical task when assessing multitasking performance in military personnel is important because military operations often involve the combination of physical and cognitive tasks, for example, being vigilant during a foot patrol or remembering the superior’s orders while running for cover.

The combination of physical and cognitive tasks can lead to the phenomenon of cognitive-motor interference, which refers to the decrement in cognitive performance and/or physical performance that occurs when a cognitive task and a physical task are performed concurrently (dual tasking) compared to when they are performed in isolation (single tasking).^4^ A considerable number of studies have investigated cognitive-motor interference while walking, showing evident gait performance impairments^5^ and higher cognitive workloads^6^ under dual-task conditions. However, less research has been conducted on other physical tasks, such as running,^7–10^ swimming,^11^ and climbing.^12^ Despite the general trend for decrements in both physical and cognitive performance, there have been reports of no significant impairments in running performance during dual tasking,^8,9^ which may suggest a potential resource prioritization in favor of physical tasks when the physical demands are substantial. Even though the mechanisms underlying cognitive-motor interference are still unclear, one of the most accepted theories in this field suggests that both tasks would rely on shared and limited resources at the brain level.^13^ Neuroimaging and electrophysiological studies have confirmed that such “resources” are neural networks that overlap across cognitive and motor tasks.^4^

With regard to tests assessing cognitive-motor interference, a team of rehabilitation scientists developed the Assessment of Military Multitasking Performance that includes physical tasks such as walking and running. The Assessment of Military Multitasking Performance is a battery of clinical dual tasks and multitasks intended to evaluate the return-to-duty requirements in the military population affected by mild traumatic brain injury.^14,15^ Some studies have also investigated cognitive interference in gait and postural stability in healthy people^16^ or following concussion and mild traumatic brain injury.^17–19^ A multimodal database aimed at assessing mental workload during physical workload has also been described.^20^ Nevertheless, more work is required to develop valid and reliable tests of cognitive-motor interference in healthy soldiers and other military personnel.

The purpose of the present study was to investigate the construct validity (conceptualized as the ability to demonstrate a significant cognitive-motor interference) and test–retest reliability of two novel cognitive-motor interference tests on soldiers, officers, and cadets free of brain injury. It was hypothesized that both tests would be valid and reliable measures of cognitive-motor interference and that decrements in both cognitive and physical performance would be found.

## METHODS

### Participants

Twenty-four soldiers, officers, and cadets (20 males and 4 females) (means ± SD: age 28.5 ± 6.0 years, body mass 76 ± 10 kg, height 1.80 ± 0.08 m, and V̇O_2max_ 53.1 ± 5.8 mL/kg/min) were recruited from the Royal School of Military Engineering (RSME) in Chatham and the Royal Air Force College (RAFC) in Cranwell. In order to be eligible for the study, participants had to be free of any illness, disability, or injury that may have precluded safe participation in vigorous exercise, any sensitivity to flashing lights (e.g., people suffering from some forms of epilepsy), and mental illness or learning disability (with the exception of mild dyslexia). All volunteers received a Participant Information Sheet and signed the Standard Consent Form before taking part in the study. Two participants did not complete the initial tests because of injury unrelated to the study and other withdrawal reasons. A further two participants were lost to follow-up because of injury and illness unrelated to the study. The study was approved by the Ministry of Defence Research Ethics Committee and conducted in conformity with the Declaration of Helsinki.

### Study Design

A test–retest design was used for this study. After a preliminary visit, participants attended four experimental visits: visit 1 and visit 2 (tests) and visit 3 and visit 4 (retests). Participants from RSME were required to attend the Physiology Laboratory at the University of Kent (Medway), whereas participants from the RAFC were asked to visit a temporary Physiology Laboratory arranged at the College. The retest visits were conducted 2 weeks after the test visits (see “Testing Procedures”). Both test and retest visits were separated by a minimum of a 48-hour recovery period and conducted over a period of 7 days. All experimental visits were performed at the same time of the day (±2 h) and completed under the same environmental conditions (temperature: 18–20 °C; humidity: 40–45%).

Participants performed a 10-min loaded marching, a 10-min Psychomotor Vigilance Task (PVT), and the two tasks combined (dual task 1) at visit 1 and visit 3; a 5-min running time trial, a 5-min Word Recall Task (WRT), and the two tasks combined (dual task 2) at visit 2 and visit 4. Step length, step frequency, mean reaction time, and number of lapses were measured at visits 1 and 3; running distance and number of words recalled at visits 2 and 4. All variables were measured in both single- and dual-task conditions. The tests were chosen as they present some face validity. Indeed, soldiers and pilots often have to perform high-intensity running and loaded marching in conditions of cognitive overload, as well as be vigilant, react quickly, and remember instructions and procedures under physiologically stressful conditions.

Subjects were instructed to maintain their normal diet throughout the testing period, to avoid food and drinks at least 1 hour before the visits, to abstain from strenuous exercise and alcohol consumption 24 hours before each testing session, and to avoid any caffeinated drink/food at least 3 hours before. Participants were also required to drink ∼35 mL of water per kilogram of body weight in the 24 hours before testing and to sleep at least 7 hours the night before testing. Before each visit, in order to verify participants' adherence to these instructions, a verbal pre-experimental checklist was completed. Visits were rescheduled if participants did not adhere to them.

### Testing Procedures

#### Preliminary visit

Following the measurement of body mass and stature, participants were asked to execute an incremental test on a motorized treadmill set at a 1% grade (RAFC: Pulse Fitness Club Line 260 G; Pulse Fitness Ltd, Cheshire, United Kingdom. RSME: Pulsar 3P; h/p/cosmos Sports and Medical, Nussdorf-Traunstein, Germany). The test was used to assess participants’ general fitness level. It started at a speed of 8 km/h with incremental increases of 1 km/h every minute until volitional exhaustion. The pulmonary gas exchange was measured breath-by-breath throughout the entire test (RAFC: COSMED Fitmate MED; Cosmed Srl, Rome, Italy. RSME: MetaLyzer 3B; Cortex Biophysik GmbH, Leipzig, Germany). Heart rate (HR) was measured at rest and during running using a telemetry monitor strapped around the chest (RAFC and RSME: Polar V800; Polar Electro Oy, Kempele, Finland). During the test, participants were also invited to rate their subjective feelings of effort using the Rating of Perceived Exertion scale developed by Borg.^21^ One minute after exercise, a 10-μL sample of whole fresh blood was taken from a fingertip and analyzed for lactate concentration (RAFC: Lactate Scout 4; EFK Diagnostics, SensLab GmbH, Leipzig, Germany. RSME: Biosen; EFK Diagnostics, London, UK). The highest 30-second moving average of V̇O_2_ measured during the incremental step test was recorded as V̇O_2max_ if a plateau in V̇O2 occurred at the end of the test or the following criteria were met: HR ≥ 95% of age-predicted maximum HR (220—age); lactate concentration ≥ 6 mmol; and Rating of Perceived Exertion ≥ 18. All participants were able to meet the above criteria. After this test, participants were familiarized with the dual-tasking tests.

#### Loaded Marching + PVT test (visits 1 and 3)

Participants were required to walk on the same motorized treadmill set at a 1% grade for 10 minutes at 5 km/h while wearing their personal rucksack (i.e., a camelbak daysack) with a weight corresponding to 30% of their body weight (task 1). The rucksack was packed by the participants using a standard military technique, adopted to prevent lower back injuries (i.e., the weight was distributed using lighter bulkier materials/items at the bottom of the rucksack and the heaviest ones at the very top). Participants were required to tie both the sternum strap and waist belt. During this loaded march, participants’ gait was analyzed for step length and frequency using an optical gait analysis system (Optogait, Microgate, Bolzano, Italy) with detectors fixed to the motorized treadmill. After 10-min rest, participants were asked to perform a standard 10-min PVT (task 2) while standing on the same but inactive motorized treadmill. The PVT is a task that measures the speed with which participants respond to visual stimuli randomly presented, and it has been widely used to assess sustained attention in soldiers and other military cohorts.^22^ Participants were instructed to respond as quickly as possible to the visual stimuli (a bullseye), which were presented randomly with an inter-stimulus interval of 1 to 10 seconds on a computer screen attached to a laptop loaded with a specific software for cognitive testing (E-prime 2.0 software, Psychology Software Tools, Inc., Pennsylvania, United States). Reaction time was recorded using a hand-held response button attached to the same laptop via a response box. After another 10-min break, participants performed task 1 and task 2 at the same time (dual task 1). PVT performance was analyzed as mean reaction time and number of lapses over the 10-min period. Gait was analyzed as mean step length and frequency over the 10-min period. Tasks’ order was randomized and counterbalanced.

#### Running Time Trial + WRT test (visits 2 and 4)

This test was based on the protocol developed by Epling and colleagues.^8^ Participants were invited to run as far as they can in 5 minutes (time trial) on the same motorized treadmill grade 1% (task 1). After a 20-min rest, they performed the WRT in a seated single-task condition (task 2). Then, they would perform the WRT while running again with the same goal of covering as much distance as possible in 5 minutes (dual task 2). Four 20-word lists from the Paivio et al.^23^ word pool were used for the WRT. The words were balanced for frequency, concreteness, imagery, and meaningfulness, each with two syllables and five to seven letters. The words were recorded by a British speaker and provided to the participants via earphones connected to a smartphone via Bluetooth. One word was played every 15 seconds so that participants were presented with 20 words in 5 minutes. At the end of this 5-min period, participants were given 90 seconds to write down all the words they remembered. The word lists and the task order were randomized and counterbalanced.

### Statistical Analysis

The Shapiro–Wilk test, histograms, Q–Q plots, and boxplots were used to check for outliers and normality of the data. The construct validity of the cognitive-motor interference tests was assessed on the data collected during visit 1 and visit 2 as they included the highest number of participants (*n* = 22). Construct validity was conceptualized as the ability to demonstrate a significant cognitive-motor interference. Statistically, construct validity was established using nonparametric Wilcoxon signed-rank tests to test the effect of task condition (single task vs. dual task) on the number of lapses (as data were not normally distributed) and paired-sample *t*-tests to test the effect of task condition on the other parameters of cognitive and physical performance. To aid in the interpretation of the significant cognitive-motor interference effects, Cohen’s d with Hedges’ correction was calculated using the SD of the difference as the standardizer (d = 0.20 small, d = 0.50 moderate, and d = 0.80 large).^24^

Reliability was assessed for each variable in each task condition (single task and dual task) using the Intraclass Correlation Coefficient (ICC) test–retest, based on a two-way mixed model effect, absolute agreement, multiple measurements, and average measures.^25^ ICC values lower than 0.5, between 0.5 and 0.75, between 0.75 and 0.9, and higher than 0.90 were classified as poor, moderate, good, and excellent reliability, respectively. Nonparametric Rothery’s ICC was used to test the reliability of the number of lapses. Lower and upper limit bounds of the 95% CIs were also reported.^25^ The Bland–Altman analysis was used to assess the agreement between test and retest for each variable in each task condition (single task and dual task). The proportionality of the bias was identified by a statistically significant slope (*P* < .05) of the regression line. Two participants were excluded from this analysis as they did not complete all the test and retest visits. One additional participant was excluded from the test–retest analysis of the loaded marching variables (i.e., step length and step frequency) because of technical issues during the data collection.

Unless otherwise stated, data are presented as mean ± SD. Significance was set at 0.05 (two tailed) for all analyses, which were conducted using the SPSS statistical package (version 24.0; SPSS, Chicago, Illinois, United States). The Rstudio software (v2022.02.1 + 461, PBC, Boston, MA, United States) and the R package “Nopaco” were used to compute the nonparametric Rothery’s ICC.^26^

## RESULTS

### Running Time Trial + WRT Test

**FIGURE 1.**
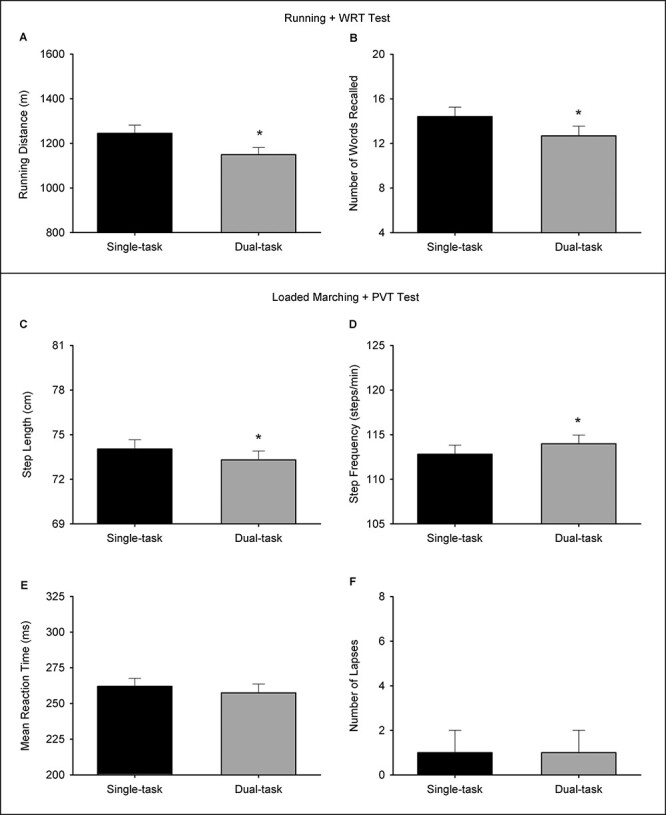
The effect of task during the Running Time Trial + WRT test: running distance (A) and number of words recalled (B). The effect of task during the Loaded Marching + PVT test: step length (C), step frequency (D), mean reaction time (E), and number of lapses (F). *n* = 22. Data are presented as mean ± SEM except for the number of lapses (F), which is displayed as median ± interquartile range. * denotes statistical significance (*P* < .05).

Paired *t*-tests revealed a significant effect of task on both running distance (*t*_(21)_ = 5.600, *P* < .001) and number of words recalled (*t*_(21)_ = 3.227, *P* = .004). The running distance was shorter in the dual-task condition (1,149 ± 154 m) compared to the single-task condition (1,245 ± 171 m). The number of words recalled was lower in the dual-task condition (13 ± 4 words) compared to the single-task condition (14 ± 4 words) (Fig. 1A and B).

### Loaded Marching + PVT Test

Paired *t*-tests revealed a significant effect of task on both step frequency (*t*_(21)_ = −4.506, *P* < .001) and step length (*t*_(21)_ = −0.721, *P* < .001) during the loaded marching. Step frequency was higher in the dual-task condition (114 ± 5 steps · min^−1^) compared to the single-task condition (113 ± 5 steps · min^−1^). Step length was shorter in the dual-task condition (73.3 ± 2.8 cm) compared to the single-task condition (74.0 ± 2.9 cm). No significant differences were found on mean reaction time (*t*_(21)_ = 0.856, *P* = .402) and number of lapses (*Z* = −0.721, *P* = .479) during the PVT (Fig. 1C–F).

### Test–Retest Reliability

**FIGURE 2.**
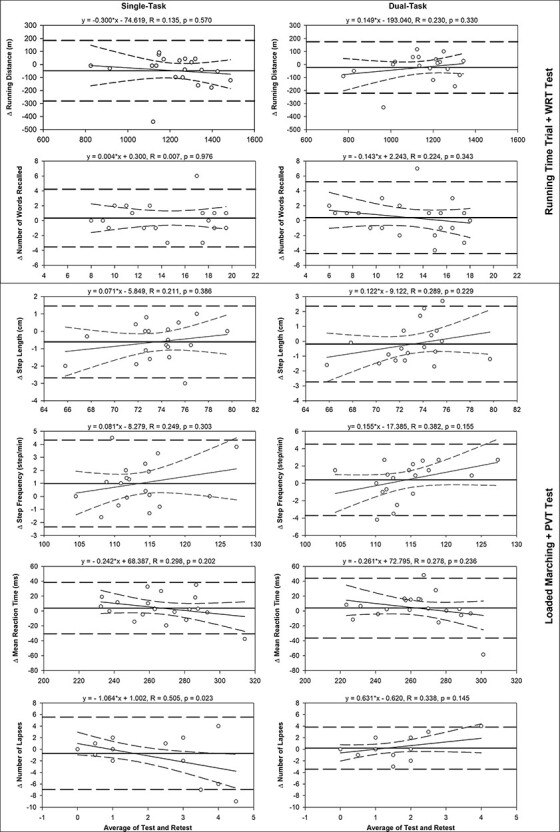
Bland–Altman plots of test–retest single-task (left column) and dual-task (right column) running distance, number of words recalled, step length, step frequency, mean reaction time, and number of lapses. The solid horizontal lines represent the mean difference (i.e., mean bias) between test and retest; the long dashed horizontal lines represent the 95% limits of agreement. Regression lines and 95% CIs (short dashed lines) are also displayed.

Good-to-excellent reliability was found for the vast majority of cognitive and physical variables in both single- and dual-task conditions (see Table I for further details). Only the number of lapses variable in the PVT showed moderate reliability in both conditions. The Bland–Altman analysis is displayed in Fig. 2.

**TABLE I.** Test–Retest Reliability (*n* = 19 for Step Length and Step Frequency; *n* = 20 for All the Other Variables)

**Running Time Trial + WRT test**						
Variable (units)	Test $\overline{x}$ ± SD	Retest $\textstyle\overline x$ ± SD	Mean difference (*P*-value)	ICC (*P*-value)	95% CI Lower bound	95% CI Upper bound
ST distance (m)	1246 ± 178	1199 ± 163	47 ± 118 (0.088)	0.849 (<.001)	0.620	0.940
DT distance (m)	1154 ± 152	1131 ± 174	22 ± 101 (0.327)	0.895 (<.001)	0.739	0.958
ST words recalled (number of words)	14.6 ± 4.1	14.9 ± 4.1	−0.3 ± 2.0 (0.439)	0.938 (<.001)	0.846	0.975
DT words recalled (number of words)	12.7 ± 4.3	13.1 ± 3.8	−0.4 ± 2.5 (0.476)	0.900 (<.001)	0.750	0.960
**Loaded Marching + PVT test**						
Variable (units)	Test $\overline{x}$ ± SD	Retest $\overline{x}$ ± SD	Mean difference (*P*-value)	ICC (*P*-value)	95% CI Lower bound	95% CI Upper bound
ST step length (cm)	73.9 ± 3.1	73.3 ± 3.3	0.6 ± 1.1 (0.021)	0.964 (<.001)	0.881	0.987
DT step length (cm)	73.2 ± 2.9	73.0 ± 3.3	0.2 ± 1.3 (0.533)	0.957 (<.001)	0.889	0.983
ST step frequency (steps/min)	113.0 ± 5.1	113.9 ± 5.5	−0.9 ± 1.7 (0.021)	0.966 (<.001)	0.887	0.988
DT step frequency (steps/min)	114.2 ± 4.8	114.6 ± 5.6	−0.4 ± 2.1 (0.411)	0.959 (<.001)	0.896	0.984
ST mean RT (ms)	265 ± 26	269 ± 21	−4 ± 18 (0.340)	0.835 (<.001)	0.591	0.934
DT mean RT (ms)	262 ± 26	265 ± 21	−4 ± 20 (0.408)	0.781 (<.001)	0.451	0.913
Variable (units)	Test Mdn (IQR)	Retest Mdn (IQR)		Rothery’s ICC (*P*-value)	95% CI Lower bound	95% CI Upper bound
ST lapses (number of lapses)	1 (2)	1 (2)		0.709 (.195)	0.631	1.000
DT lapses (number of lapses)	1 (2)	1 (2)		0.698 (.257)	0.610	1.000

Abbreviations: WRT, Word Recall Task; $\overline{x}$, mean; ICC, Intraclass Correlation Coefficient; ST, single task; DT, dual task; PVT, Psychomotor Vigilance Test; RT, reaction time; Mdn, median; IQR, interquartile range.

## DISCUSSION

### Validity

The current study has provided initial evidence that these novel dual-tasking tests are valid measures of cognitive-motor interference in military contexts. Specifically, strong evidence of cognitive-motor interference was found during the Running + WRT test, where both cognitive and physical performances were impaired in the dual-task condition. The moderate cognitive-motor interference effect on short-term memory (number of words recalled d = −0.68) and the large cognitive-motor interference effect on endurance performance (running distance d = −1.17) are larger than the negative effects of sleep deprivation.^27,28^ Given the well-established negative impact of sleep deprivation on soldier performance, such comparisons suggest that the cognitive-motor interference effects measured in our study are not trivial.

Our results are in partial agreement with previous studies conducted in athletes and physically active individuals, which showed significant impairments in WRT performance but no significant decrements in self-paced running performance during dual tasking.^8,9^ This different outcome may be because of two main factors. First, Epling and colleagues^8^ conducted the experiment on a 400-m track and, consequently, participants may have had some awareness of the distance covered. The 5-min running time trial performed in the present experiment was performed on a motorized treadmill, and no feedback about the distance covered was given. Some awareness of the distance covered might have facilitated performance and therefore decreased the interference of WRT on the running task in Epling and colleagues’ study. Second, it is possible that manual regulation of the treadmill speed is more cognitively demanding than the more natural pace regulation that occurs on the track.

The evidence for the construct validity of the Loaded Marching + PVT test is weaker because significant cognitive-motor interference was found for physical performance only. These findings are in contrast with a previous study conducted on healthy young adults, which showed significant reaction time decrements during a visuomotor reaction time task performed while walking.^29^ Considering that military personnel generally have higher levels of fitness and multitasking skills, it is likely that the loaded marching used in our study was not demanding enough. Increments in loading weight and/or marching speed may enhance the physical demand during the PVT. In order to improve the construct validity of this test in terms of cognitive performance, it might also be useful to increase the difficulty of the cognitive task itself by adding some internal interfering factors, such as working memory and mental tracking (i.e., holding information while carrying out mental operations).^30^ Indeed, it was shown that these factors generate a greater interference on gait performance than simple reaction time, which is considered an external interfering factor.^5,29^ Virtual reality environments might also be used to increase in a more ecological way the cognitive demands of loaded marching by simulating military-relevant cognitive tasks such as the identification of visual cues related to the presence of improvised explosive devices along the track.

With regard to physical performance, a significant reduction in step length and a significant concomitant increase in step frequency were observed in the dual-task condition compared to the single-task condition. Both of these cognitive-motor interference effects were large (step frequency d = 0.94 and step length d = −0.83). Alterations in step length and step frequency have previously been shown when a cognitive task is added to walking in healthy populations,^5^ and our study shows that trained military personnel are not immune from the negative effects of cognitive-motor interference on motor control. Furthermore, persistent cognitive-motor interference effects on gait may lead to musculoskeletal injuries in active individuals.^31^

### Reliability

The test–retest results indicate that the Running + WRT test can be considered a reliable cognitive-motor interference test for assessing multitasking performance in military contexts. Good (ICC between 0.75 and 0.9) to excellent (ICC > 0.9) reliability was shown during both single and dual tasks in the running distance and number of words recalled variables, respectively. Only the number of lapses in the PVT showed moderate reliability (ICC between 0.5 and 0.75). Furthermore, there were order effects (i.e., changes over time) in both step length and step frequency, showing a learning effect between the first and second loaded march on the motorized treadmill, which may be reduced further by participant familiarization. Consequently, this test needs further investigation and possible modifications. Moreover, the development of other and more specific cognitive-motor performance tests (e.g., flight simulation during exercise on a reclined cycle ergometer to induce physiological stress) should also be considered.

## CONCLUSION AND PRACTICAL APPLICATIONS

The current study provides initial evidence that the Running + WRT test is a valid and reliable dual-tasking test and that it could be used to assess cognitive-motor interference in soldiers, officers, and cadets. On the other hand, the Marching + PVT test requires further work to enhance its validity and reliability, especially in terms of cognitive performance. From a practical perspective, the Running + WRT test may be used to assess the multitasking performance of both soldiers and pilots and monitor any potential improvement induced by training. However, further research is required to assess its sensitivity to change and, if possible, to increase its ecological validity by, for example, having participants memorize military-relevant instructions rather than just simple words.

It is important to highlight that the current study can only provide evidence related to the construct validity of the dual-tasking tests by showing that they are difficult enough to induce cognitive-motor interference in trained and healthy military personnel. It would be useful to establish to what extent performance in these relatively simple dual tasks correlates with the performance of more complex military tasks like, for example, following instructions about tactics to implement immediately on a battlefield while engaging in the protective physical activities needed when taking fire. However, this kind of test validation (criterion-related validity) is not possible because, to date, there are no well-validated and reliable measures of multitasking performance in simulated battlefield environments. We hope that the present study will stimulate future work, leading to the development and validation of such criterion measures as well as further validation of the simpler dual-tasking tests reported here.

## Data Availability

The data underlying this article will be shared on reasonable request to the corresponding author.

## INSTITUTIONAL CLEARANCE

Institutional clearance approved.

## References

[1] Chérif L , Wood V, Marois A, Labonté K, Vachon F: Multitasking in the military: cognitive consequences and potential solutions. Appl Cogn Psychol 2018; 32(4): 429–39.doi: 10.1002/acp.3415.

[2] Dehais F , Causse M, Vachon F, Régis N, Menant E, Tremblay S: Failure to detect critical auditory alerts in the cockpit: evidence for inattentional deafness. Hum Factors 2014; 56(4): 631–44.doi: 10.1177/0018720813510735. 25029890

[3] Santiago-Espada Y , Myer RR, Latorella KA: The Multi-Attribute Task Battery II (MATB-II) software for human performance and workload research: a user’s guide (NASA/TM-2011–217164) [published online 2011]. Hampton, VA: National.

[4] Leone C , Feys P, Moumdjian L, D’Amico E, Zappia M, Patti F: Cognitive-motor dual-task interference: a systematic review of neural correlates. Neurosci Biobehav Rev 2017; 75: 348–60.doi: 10.1016/j.neubiorev.2017.01.010. 28104413

[5] Al-Yahya E , Dawes H, Smith L, Dennis A, Howells K, Cockburn J: Cognitive motor interference while walking: a systematic review and meta-analysis. Neurosci Biobehav Rev 2011; 35(3): 715–28.doi: 10.1016/j.neubiorev.2010.08.008. 20833198

[6] Hoang I , Ranchet M, Derollepot R, Moreau F, Paire-Ficout L: Measuring the cognitive workload during dual-task walking in young adults: a combination of neurophysiological and subjective measures. Front Hum Neurosci 2020; 14: 592532.doi: 10.3389/fnhum.2020.592532. PMC7714906 33328938

[7] Blakely MJ , Kemp S, Helton WS: Volitional running and tone counting: the impact of cognitive load on running over natural terrain. IIE Trans Occup Ergon Hum Factors 2016; 4(2–3): 104–14.doi: 10.1080/21577323.2015.1055864.

[8] Epling SL , Blakely MJ, Russell PN, Helton WS: Free recall and outdoor running: cognitive and physical demand interference. Exp Brain Res 2016; 234(10): 2979–87.doi: 10.1007/s00221-016-4700-y. 27299913

[9] Epling SL , Edgar GK, Russell PN, Helton WS: How does physical demand affect cognitive performance? Interference between outdoor running and narrative memory. Proc Hum Fact Ergon Soc Annu Meet 2018; 62(1): 237–41.doi: 10.1177/1541931218621055.

[10] Blakely MJ , Wilson K, Russell PN, Helton WS: The impact of cognitive load on volitional running. Proc Hum Fact Ergon Soc Annu Meet 2016; 60(1): 1179–83.doi: 10.1177/1541931213601276.

[11] Stets A , Smith SL, Helton WS: Dual-task interference between swimming and verbal memory. *Hum Factors* 2020; 62(7): 1132–40.doi: 10.1177/0018720819871743. 31513440

[12] Woodham A , Billinghurst M, Helton WS: Climbing with a head-mounted display: dual-task costs. Hum Factors 2016; 58(3): 452–61.doi: 10.1177/0018720815623431. 26865416

[13] Wickens CD , Tsang PS, Benel RA: The allocation of attentional resources in a dynamic environment. Proc Hum Fact Soc Ann Meet 1979; 23(1): 527–31.doi: 10.1177/1071181379023001131.

[14] Radomski MV , Weightman MM, Davidson LF, et al: Development of a measure to inform return-to-duty decision making after mild traumatic brain injury. Mil Med 2013; 178(3): 246–53.doi: 10.7205/MILMED-D-12-00144. 23707109

[15] Weightman MM , McCulloch KL, Radomski MV, et al: Further development of the assessment of military multitasking performance: iterative reliability testing. PLoS One 2017; 12(1): e0169104.doi: 10.1371/journal.pone.0169104. PMC5215871 28056045

[16] Penko AL , Linder SM, Miller Koop M, Dey T, Alberts JL: Quantification of dual-task performance in healthy young adults suitable for military use. Mil Med 2021; 186(Suppl 1): 58–64.doi: 10.1093/milmed/usaa404. 33499500

[17] Catena RD , van Donkelaar P, Chou LS: Cognitive task effects on gait stability following concussion. Exp Brain Res 2007; 176(1): 23–31.doi: 10.1007/s00221-006-0596-2. 16826411

[18] Catena RD , van Donkelaar P, Chou LS: Altered balance control following concussion is better detected with an attention test during gait. Gait Posture 2007; 25(3): 406–11.doi: 10.1016/j.gaitpost.2006.05.006. 16787746

[19] Chou LS , Kaufman KR, Walker-Rabatin AE, Brey RH, Basford JR: Dynamic instability during obstacle crossing following traumatic brain injury. Gait Posture 2004; 20(3): 245–54.doi: 10.1016/j.gaitpost.2003.09.007. 15531171

[20] Albuquerque I , Tiwari A, Parent M, et al: WAUC: a multi-modal database for mental workload assessment under physical activity. Front Neurosci 2020; 14: 549524.doi: 10.3389/fnins.2020.549524. PMC7736238 33335465

[21] Borg G : *Borg’s Perceived Exertion and Pain Scales*. Human Kinetics; 1998.

[22] McLellan TM , Kamimori GH, Bell DG, Smith IF, Johnson D, Belenky G: Caffeine maintains vigilance and marksmanship in simulated urban operations with sleep deprivation. Aviat Space Environ Med 2005; 76(1): 39–45. 15672985

[23] Paivio A , Yuille JC, Madigan SA: Concreteness, imagery, and meaningfulness values for 925 nouns. J Exp Psychol 1968; 76(1): 1–25.doi: 10.1037/h0025327. 5672258

[24] Lakens D : Calculating and reporting effect sizes to facilitate cumulative science: a practical primer for t-tests and ANOVAs. Front Psychol 2013; 4: 863.doi: 10.3389/fpsyg.2013.00863. PMC3840331 24324449

[25] Koo TK , Li MY: A guideline of selecting and reporting intraclass correlation coefficients for reliability research. J Chiropr Med 2016; 15(2): 155–63.doi: 10.1016/j.jcm.2016.02.012. 27330520 PMC4913118

[26] Kuiper R , Hoogenboezem R, Huisman S, Sonneveld P, van Duin M: nopaco: a non-parametric concordance coefficient. R Package Version 2019; 1(6).

[27] Lim J , Dinges DF: A meta-analysis of the impact of short-term sleep deprivation on cognitive variables. Psychol Bull 2010; 136(3): 375–89.doi: 10.1037/a0018883. 20438143 PMC3290659

[28] Lopes TR , Pereira HM, Bittencourt LRA, Silva BM: How much does sleep deprivation impair endurance performance? A systematic review and meta-analysis. *Eur J Sport Sci*. Published online ahead of print 6 December 2022.doi: 10.1080/17461391.2022.2155583. 36472094

[29] Patel P , Lamar M, Bhatt T: Effect of type of cognitive task and walking speed on cognitive-motor interference during dual-task walking. Neuroscience 2014; 260: 140–8.doi: 10.1016/j.neuroscience.2013.12.016. 24345478

[30] Lezak MD , Howieson DB, Loring DW, Fischer JS: *Neuropsychological Assessment*. Oxford University Press; 2004.

[31] Howell DR , Bonnette S, Diekfuss JA, et al: Dual-task gait stability after concussion and subsequent injury: an exploratory investigation. Sensors 2020; 20(21):6297.doi: 10.3390/s20216297. PMC7663806 33167407

